# Diagnostic efficacy of CCTA and CT-FFR based on risk factors for myocardial ischemia

**DOI:** 10.1186/s13019-022-01787-w

**Published:** 2022-03-19

**Authors:** Gao Yongguang, Shi Yibing, Xia Ping, Zhang Jinyao, Fu Yufei, Huang Yayong, Xu Yuanshun, Li Gutao

**Affiliations:** 1 Department of Radiology, Xuzhou Central Hospital, 199 Jiefang Road, Xuzhou, Jiangsu China; 2 Beijing Keyaark Medical Technology Co., Ltd, Beijing, China; 3 Graduate School, Bengbu Medical College, 2600 Donghai Avenue, Bengbu, Anhui China

**Keywords:** CCTA, CT-FFR, Myocardial ischemia, Fractional flow reserve

## Abstract

**Background:**

Coronary artery coronary computed tomography angiography (CCTA) can observe the degree of coronary artery stenosis and fractional flow reserve (FFR) can diagnose hemodynamic abnormalities caused by coronary artery stenosis. However, noninvasive imaging examination that can both observe the above two methods at the same time has not yet been elucidated.

**Objective:**

To investigate the diagnostic efficacy of CCTA and computed tomography-derived fractional flow reserve (CT-FFR) based on different risk factors for myocardial ischemia.

**Methods:**

Patients undergoing CCTA in our hospital from August 18, 2020 to April 28, 2021 were randomly selected, and the data were subjected to CT-FFR analysis. Vascular characteristics were measured, including total plaque volume, calcified plaque volume, non-calcified plaque volume, plaque length, and lumen stenosis, and the patients were categorized into a non-ischemia group (FFR > 0.8) and an ischemia group (FFR ≤ 0.8). Plaque characteristics were compared between the two groups, and logistic regression analysis was employed to explore the correlations between plaque characteristics and ischemic lesions.

**Results:**

From a total of 122 patients enrolled in the study, there were 218 vascular branches with FFR > 0.8 and 174 vascular branches with FFR ≤ 0.8. There were significant group differences in total plaque volume, calcified plaque volume, plaque length, and lumen stenosis > 50% (n). The obtained data were as follows: non-ischemic group 10.57 (4.80, 259.65), ischemic group 14.87 (3.39, 424.45), *Z* = 9.772, *p* = 0.002, non-ischemic group 10.57 (0, 168.77), ischemic group 14.87 (0, 191.00), *Z* = 2.503, *p* ≤ 0.001), non-ischemic group 8.17 (37.05, 40.53), ischemic group 8.38 (56.66, 86.47), *Z* = 5.923, *p* = 0.016, and lumen stenosis > 50%, non-ischemic group 46, ischemic group 90, *x*^2^ = 14.77, *p* ≤ 0.001. The regression analysis results indicated that total plaque volume, calcified plaque volume, plaque length and lumen stenosis > 50% were risk factors for myocardial ischemia, with ORs and *p* values of (2.311, *p* = 0.002), (1.021, *p* = 0.004), (2.159, *p* < 0.001), and (0.181, *p* < 0.001), respectively.

**Conclusion:**

Total plaque volume, calcified plaque volume, plaque length and lumen stenosis > 50% are predictors for myocardial ischemia. Coronary artery CCTA combined with CT-FFR could simultaneously observe the anatomical stenosis and evaluate myocardial blood supply at the functional level. Thus, myocardial ischemia could be better diagnosed.

## Background

China has an immense population and a substantial number of people with coronary atherosclerosis. Coronary computed tomography angiography (CCTA) is a first-line imaging tool to exclude and diagnose coronary heart disease, and is characterized by low cost, simplicity of operation, and high negative predictive value. CCTA has been used extensively as a non-invasive approach to determine coronary heart disease in China.

CCTA can identify anatomical stenosis, but cannot functionally evaluate hemodynamic abnormalities caused by coronary artery stenosis. FFR relying on invasive coronary angiography (ICA) observes hemodynamic abnormalities, but it is difficult to promote widely due to its invasive nature (e.g., possible destruction of plaque stability during surgery, or vascular wall damage), high cost, increased surgical risk, and adenosine side effects. However, CT-derived FFR (CT-FFR) is emerging as a non-invasive method to solve these problems, which can both identify vascular anatomical stenosis and observe hemodynamic abnormalities.

With the rapid progression of AI and electronic computers, non-invasive CT‑FFR has developed rapidly in recent years. Previous studies have confirmed that CT-FFR is highly accurate in diagnosing coronary artery stenosis [1, 2]. Moreover, CT-FFR was highly correlated with traditional ICA-FFR, since the relevant outcomes can be calculated using the basic governing equation of fluid mechanics, namely the Navier–Stokes equation which assumes that blood is an incompressible Newtonian fluid with constant density and viscosity [3]. In 2019, according to *Guidelines for the Diagnosis and Management of Chronic Coronary Syndromes* released by the European Society of Cardiology, non-invasive functional imaging techniques have been recommended to be first-line examination approaches (Classification I recommendation) for clinical diagnosis and treatment of patients with suspected or known coronary heart disease [4].

An ICA-based diagnosis alone is not sufficient in the case of partial diseased vascular stenosis > 50%. The reason for this is that some diseased blood vessels may form collaterals, resulting in a certain compensatory effect, and ICA analysis cannot truly detect the myocardial ischemia dominated by the diseased blood vessels, leading to a mismatch between stenosis severity and myocardial ischemia [5]. Studies have demonstrated that for patients who are considered for stent implantation after ICA examination, approximately 32% do not require stent intervention [6]. Therefore, the search for a better tool to judge functional coronary artery stenosis has become a current research hotspot.

## Objectives

In view of a substantial number of people with coronary atherosclerosis in China, the present study aims to investigate the influences of different risk factors for myocardial ischemia on CT-FFR using CCTA, so as to allow more atherosclerosis patients to receive the benefits of imaging examination, such as low cost and non-invasiveness.

## Methods

### General data

The clinical data of 132 patients diagnosed with known or suspected coronary heart disease after undergoing CCTA were retrospectively analyzed by CT-FFR. The inclusion criteria were as follows: (a) patients with qualified CCTA image quality, and (b) those with valid data following CT-FFR software calculation and feedback. Patients were excluded for the following: (a) having received a stent implantation (n = 4), (b) having undergone coronary artery bypass grafting surgery (n = 4), and (c) having data that could not be identified by DICOM software (n = 2). A total of 122 patients were enrolled, with 396 diseased blood vessels, including 218 vascular branches with FFR > 0.8 and 174 vascular branches with FFR ≤ 0.8, 168 anterior descending branches, 56 circumflex branches, 120 right coronary arteries, 16 first diagonal branches, 10 s diagonal branches, 10 blunt marginal branches, and 12 intermediate branches, which were subjected to CT-FFR plaque measurement and evaluation. Among the 122 patients, there were 72 males and 50 females with ages ranging from 32 to 82 (59.9 ± 8.6) years old.

### Research methods

All patients received conventional CCTA according to the *Society of Cardiovascular Computed Tomography Guidelines*. In this study, two CT scanners were used, a 320-row CT (Aquilion One, Toshiba, Japan) and a 640-row CT (Aquilion ViSION, Toshiba, Japan). A double-headed high-pressure syringe was utilized to inject 40–60 mL of the non-ionic contrast agent iopromide (containing 370 mg/mL iodine, Bayer Schering Pharma) at a flow rate of 4.0–5.0 mL/s, and then 30 mL of normal saline at the same flow rate. Patients were in the supine position during the scan and were routinely monitored by electrocardiograph (ECG). The scan range was 1.0 cm below the carina to 1.5 cm from the inferior margin of the heart. No patients received nitroglycerin or β-blockers. CT scan parameters included: (a) collimation: 320 × 0.500, reconstruction thickness: 0.500 mm, tube rotation speed: 0.35 s, tube voltage: 100 kV or 120 kV, tube current: 200–400 mA, and detector collimation: 16–320 × 0.5 mm, and (b) Aquilion ViSION, scan parameters: collimation: 160–320 × 0.5 mm, reconstruction thickness: 0.500 mm, tube rotation speed: 0.275 s/r, tube voltage: 100 kV (smart), and tube current: 200–400 mA. All scanning devices adopt prospective ECG gating for image acquisition, followed by optimal image quality reconstruction. The images meeting the diagnostic requirement were transferred to the post-processing workstation for subsequent analysis.

CCTA images of all enrolled patients were uploaded to the core laboratory for blind reading. Lumen stenosis on CCTA images was diagnosed by two readers with more than 5 years of working experience in coronary artery CT using the visual diameter method.

Non-invasive CT-FFR measurement was carried out as follows: the standard DICOM format CT data of all patients were transferred to the professional CT-FFR measurement software (China Keya Medical Corporation-Deep Pulse Score DEEPVESSEL FFR, China), and CT-FFR measurement was performed by a dedicated operator blinded to the results of other examinations. Moreover, all data in this study were measured twice after an interval of one month to evaluate their repeatability. Using plague segmentation and a quantitative analysis model based on deep learning algorithms, artificially labeled plaque data were processed by automatic detection and segmentation model training, and plaque volume, plaque composition, plaque length, and remodeling index of the segmentation results were calculated.

### Statistical analysis

SPSS 17.0 software was used for statistical analysis. Continuous variables were tested to ensure normal distribution. Normally distributed data were expressed by mean ± standard deviation (*χ* ± *s*), and those not conforming to a normal distribution were represented by the median (upper and lower quartiles) [M (Q1, Q2)]. Categorical data were expressed by frequency (positive remodeling, stenosis > 50%), and measurement data (total plaque volume, calcified plaque volume, non-calcified plaque volume, and plaque length) were non-normally distributed. According to the FFR value, the patients were grouped into a non-ischemia group (FFR > 0.8) and an ischemia group (FFR ≤ 0.8), and plaque characteristics were compared between the two groups. Logistic regression analysis was used to confirm the correlations between plaque characteristics and ischemic lesions. Group differences in normally distributed variables were analyzed using independent-samples *t*-tests, and Rank sum tests were utilized for non-normal variable. Data analysis was based on the number of blood vessels. *p* < 0.05 was used to indicate statistical significance.

## Results

### Comparison of clinical data

A total of 122 patients were enrolled in this study, including 66 cases with FFR > 0.8, 56 cases with FFR ≤ 0.8, 218 diseased vessels with FFR > 0.8, 174 diseased vessels with FFR ≤ 0.8 (72 male patients and 50 female patients). Regarding comorbidities, 60 patients had hypertension, 38 had diabetes, 34 had hyperlipidemia, 42 were smokers, and 48 were excessive drinkers. Group differences in the general data were not statistically significant (Table 1).

**Table 1:** Comparison of general data of patients

			Gender			Hypertension		Diabetes		Hyperlipidemia		Smoking		Drinking	
Group	n	Age (Y)□ *x* ± *s*	Male	Female	BMI kg/m^2□^ *x* ± *s*	Yes	No	Yes	No	Yes	No	Yes	No	Yes	No
FFR > 0.8	66	59.7 ± 9.1	40	26	26.3 ± 3.9	32	34	16	50	10	56	18	48	26	40
FFR ≤ 0.8	56	61.3 ± 7.7	32	24	24.9 ± 3.9	28	28	22	34	24	32	26	30	22	34
*x*^2^ (*t*)		− 0.437^a^	0		0.925^a^	0		0		0.872		0.019		0	
*p*		0.665	1		0.391	1		1		0.35		0.891		1	

FFR means fractional flow reserve. ^a^ represents *t* value

Plaque characteristics were compared between the ischemia and non-ischemia groups, and statistically significant differences were observed for total plaque volume, calcified plaque volume, plaque length, and lumen stenosis > 50% (*p* < 0.05). Patients with stenosis > 50% were common, showing a statistically significant difference. However, non-calcified plaque volume and positive remodeling were not statistically significant between the two groups (Table 2).

**Table 2:** Comparison of plaque characteristics of diseased vessels between FFR > 0.8 group and FFR ≤ 0.8 group

Group		Diseased vessels (branches)	Total volume of plaques	Volume of calcified plaques			Volume of non-calcified plaques Volume of non-calcified plaques	Lesion length	Positive remodeling (n)	Stenosis > 50% (n)
FFR > 0.8		218	10.57 (4.80, 259.65)	10.57 (0, 168.77)			32.89 (4.35, 192.41)	8.17 (37.05, 40.53)	202	46
FFR ≤ 0.8		174	14.87 (3.39, 424.45)	14.87 (0, 191.00)			43.53 (3.25, 374.35)	8.38 (56.66, 86.47)	164	90
Statistics			9.772^a^	2.503^a^			11.204^a^	5.923^b^	0^b^	14.77^b^
*p*			0.002	< 0.001			0.115	0.016	1	< 0.001

^a^Means *Z* value, and ^b^represents *χ*^2^ value

The results of logistic univariate regression analysis indicated that total plaque volume, calcified plaque volume, plaque length, and lumen stenosis > 50% could induce myocardial ischemia, with odds ratios (OR) of 2.161, 1.041, 1.322, 0.233 and *p* values of 0.002, 0.001, < 0.001, < 0.001, respectively. Risk factors showing significant differences (*p* < 0.05) were incorporated into a multivariate regression analysis, which yielded ORs of 2.311, 1.021, 2.159, 0.181, and *p* values of 0.002, 0.004, 0.002, < 0.001, respectively. Among these, lumen stenosis > 50% was the first major factor, and the OR was 0.181, *p* < 0.001 (Table 3).

**Table 3:** Regression analysis of the relationship between plaque characteristics and ischemic lesions

Index	Univariate analysis			Multivariate analysis		
	OR (95%CI)		*p*	OR (95%CI)		*p*
Total volume of plaques	2.161	1.496–4.689	0.002	2.311	2.117–5.854	0.002
Volume of calcified plaques	1.041	1.010–1.073	0.001	1.021	0.989–1.055	0.004
Volume of non-calcified plaques	0.996	0.990–1.002	0.812	–	–	–
Lesion length	1.322	1.116–1.121	< 0.001	2.159	1.482–3.385	0.002
Positive remodeling	1.175	0.357–3.864	0.79	–	–	–
Medium-severe stenosis	0.233	0.094–0.577	< 0.001	0.181	0.057–0.578	< 0.001

– Represents no data

## Discussion

According to the results of CCTA and CT-FFR, the patients were categorized into non-ischemia group (FFR > 0.8) and ischemia group (FFR ≤ 0.8). Plaque characteristics were compared between the two groups, and the results showed that the differences in total plaque volume, calcified plaque volume, plaque length, and lumen stenosis > 50% were significantly different. Moreover, different risk factors were investigated by logistic regression analysis, and the results indicated that total plaque volume, calcified plaque volume, plaque length, and lumen stenosis > 50% were influencing factors for myocardial ischemia, especially lumen stenosis > 50%, with an OR of 0.181 (*p* < 0.001). Therefore, CCTA combined with CT-FFR has high diagnostic efficacy for coronary heart disease. A routine test can be used to observe anatomical stenosis and functional test. It is noninvasive and low-cost, which reduces the burden for patients.

Multiple studies demonstrated that CT-FFR has a higher diagnostic accuracy for coronary heart disease than traditional CCTA [7–9]. Studies have confirmed that about half of the patients with significant coronary stenosis on CCTA have lumen stenosis > 50%, but without myocardial ischemia or only small patchy perfusion ischemia as detected by radionuclide imaging [10], and some patients receiving stent implantation have no significant symptom improvement after surgery [11]. In this study, the results also showed that the patients with lumen stenosis > 50% had CT-FFR > 0.8, without obvious clinical symptoms. The reason for this may be that the CT-FFR value was not decreased due to collateral formation, while patients with mild stenosis (< 50%) may suffer from myocardial ischemia, indicating that the anatomical stenosis does not match the actual functional ischemia. In most studies, FFR ≤ 0.8 is selected as the threshold, and FFR is considered as a guide [12]. When FFR > 0.8, there is no myocardial ischemia, so the therapeutic effect of medication is better. However, when FFR < 0.8, suggesting the presence of myocardial ischemia, the probability of the end-point event of patients with PCI treatment is significantly lower than that of patients with drug treatment [13]. Moreover, a portion of patients with CT-FFR slightly greater than 0.8 can also have more obvious clinical symptoms. This may be due to the fact that CT-FFR is a computer-derived data product, which has a higher correlation compared with the real-time data of invasive FFR but has differences, especially for patients with cut-off values.

The *Chinese Guidelines for Percutaneous Coronary Intervention* (2016) recommends therapeutic intervention when coronary artery stenosis is less than 90% and there is evidence of myocardial ischemia, or when FFR is less than 0.8, thereby greatly reducing the stent implantation rate and avoiding excessive medical treatment [14]. For patients with myocardial ischemia, surgical revascularization is the first choice by clinicians [15, 16]. By observing patient's clinical symptoms and evaluating stenosis severity, FFR-guided CABG has a significantly lower mortality rate or probability of myocardial infarction after 6 years over ICA-guided coronary artery bypass grafting surgery [17].

Different types of atherosclerotic plaques have different stability. The pathogenic process is the vascular intima with reactive hyperplasia evolving into fibrous and calcified plaques [18]. It is generally believed acute cardiovascular events mainly occur due to the rupture or erosion of high-risk plaques, and lumen stenosis caused by fibrous or calcified plaques can induce chronic myocardial ischemia [19]. Consistent with previous studies, risk factors such as positive remodeling, napkin-ring sign, punctate calcification, and low-density plaque are characteristic of high-risk plaques, which can increase the occurrence of acute coronary syndromes. In contrast, simple calcified and fibrous plaques can only induce myocardial ischemia due to their stable nature (i.e., not easy to rupture).

To date, CT-FFR research has made considerable progress. However, CCTA image-based CT-FFR still has many uncertain factors for blood flow and arterial blood analysis, and the reasons are as follows: (A) Different companies have different algorithms and varying calculation times. Although the processing time has shortened in recent years, it still takes a few hours at the fastest speed. (B) The research data are relatively homogeneous. The main purpose of CCTA is to screen a large range of people, but there is no relevant evidence for patients with acute coronary syndrome. (C) The CT-FFR algorithm simulates the filling state of blood vessels, which have individual differences, such as elasticity. The same model and formula will reduce accuracy, but for patients with microcirculation, the obtained CT-FFR value is too large [20]. (D) For evaluating patients with diffuse calcification, severe calcification will inevitably cause artifacts due to the limitation of CCTA images, and the data obtained will be lower.

In recent years, with the rapid development of CT-FFR, numerous experiments and evidence-based medicine verified that the technique has become the “gatekeeper” of ICA examination. ICA can characterize lumen stenosis, but cannot detect physiological ischemia. As a result, clinically significant lesions cannot be accurately treated and medical resources are wasted, while real lesions leading to myocardial ischemia go untreated [21].

There were many shortcomings in this study. Firstly, FFR was not used as the gold standard, but the accuracy of CT-FFR in diagnosing coronary artery stenosis has been confirmed in many previous studies. Moreover, CT-FFR had high correlation with traditional FFR [1–3], and the recognition was gradually improved. Secondly, this article was a retrospective study with a small sample size, and branch vessels were included in the study, which may lead to statistical biases against previous studies. Thirdly, patients who underwent stent implantation and coronary artery bypass grafting surgery were excluded from this study, so the research population needs to be comprehensively improved in future studies.

## Conclusion

In conclusion, total plaque volume, calcified plaque volume, plaque length, and stenosis lumen > 50% are risk factors for myocardial ischemia, and CCTA combined with CT-FFR can effectively diagnose myocardial ischemia.

## Data Availability

All data generated or analyzed during this study are included in this article.
